# COMPARE Family (Children of Mentally Ill Parents at Risk Evaluation): A Study Protocol for a Preventive Intervention for Children of Mentally Ill Parents (Triple P, Evidence-Based Program That Enhances Parentings Skills, in Addition to Gold-Standard CBT With the Mentally Ill Parent) in a Multicenter RCT—Part II

**DOI:** 10.3389/fpsyt.2019.00054

**Published:** 2019-02-22

**Authors:** Markus Stracke, Kristin Gilbert, Meinhard Kieser, Christina Klose, Johannes Krisam, David D. Ebert, Claudia Buntrock, Hanna Christiansen

**Affiliations:** ^1^Department of Clinical Child and Adolescent Psychology, Philipps University Marburg, Marburg, Germany; ^2^Institute of Medical Biometry and Informatics, University of Heidelberg, Heidelberg, Germany; ^3^Department of Psychology, Chair for Clinical Psychology and Psychotherapy, Friedrich-Alexander University Erlangen-Nuremberg, Erlangen, Germany; ^4^Department of Clinical, Neuro- & Developmental Psychology, Vrije Universiteit Amsterdam, Amsterdam, Netherlands

**Keywords:** children of mentally ill parents, transgenerational transmission, randomized controlled trial, prevention, intervention, mental disorders, parenting training

## Abstract

**Background:** Mental health problems are highly frequent, as well as being associated with enormous societal and economic costs and significant disability-adjusted life years. Children of parents with a mental illness (COPMI) are at a tremendously increased risk to develop disorders themselves. According to the literature, parental mental disorders launch a wave of risk factors that in turn predict the emergence of psychological problems in the offspring, and effective treatment of the parental disorder has been associated with reduced child psychopathology (launch and grow assumption). Furthermore, studies focusing on parent-child interaction demonstrate generally poorer parenting skills in parents with mental disorders, and the enhancement of such skills has been a significant mediator in improving child outcomes (parenting assumption).

**Objective:** To implement a preventive intervention for COPMI with the aim of interrupting the transmission of mental disorders in children of a parent with mental disorders. An RCT will compare state-of-the-art cognitive behavioral therapy (CBT) for a parent with mental disorders to CBT plus the Positive Parenting Program (Triple-P), a well-established and evidence-based program that enhances parenting skills.

**Methods:** A total of 634 patients seeking treatment in 8 outpatient clinics in Germany and their children will be included between January 2018 and April 2021 in the study. We use (clinical) interviews and self- as well as other-report questionnaires to assess the families at four main measurement points [T1: beginning of waiting period for psychotherapy treatment (duration of waiting period depends on usual waiting period in the study center: multiple baselines), T2: begin of parental psychotherapy, T3: post-assessment, T4: 6 months follow-up]. The total observation period will be 39 months. The patients will be randomly assigned to either the control condition (25 to 45 CBT sessions) or the experimental condition (25 to 45 CBT sessions + 10 Triple-P sessions). For evaluating the treatment process, the patients and clinicians will also be assessed after each treatment session. Furthermore, there will be a continuous assessment and report of adverse events during treatment.

**Discussion:** This trial will be the first ever to address the launch and grow as well as the parenting assumption in one study and to establish effects of the two different interventions on children's health. Our study will also likely be the first one to provide data on the comparative cost-effectiveness and will therefore provide essential information relevant for the potential implementation of such programs. The structure of the RCT will allow us to establish effects of the parental disorder(s) with/without comorbidities on children's health, to test assumptions of the trans-generational transmission model of mental disorders and bi-directional influences of different treatments on the model and to analyze specific transmission mechanisms. A deeper understanding of risk mechanisms will reveal specific transmission profiles that will result in the early detection of and effective reduction in risk factors and thus improve the health of the children at risk.

**Ethics:** The study is carried out according to the Good Clinical Practice (GCP) guidelines, the Declaration of Helsinki and its later supplements and local legal requirements. The lead ethics committee at the department of psychology at Philipps-University Marburg approved the study procedure and all study documents. A positive ethics committee vote is required at a study site, before the inclusion of a first patient at the respective site.

**Dissemination:** Via peer-reviewed publications in scientific journals, the results of this study will be made available to the scientific community. Using PsychData all primary data will be made available for re- and meta-analyses. Politicians, public health services, and stakeholders will be informed throughout the study and beyond, thus, improving public policy and health care decisions concerning preventive interventions and treatments for COPMI.

**Trial Registration:** DRKS-ID: DRKS00013516 (German Clinical Trials Register, https://www.drks.de/drks_web/navigate.do?navigationId=trial.HTML&TRIAL_ID=DRKS00013516)

## Introduction

Children of mentally ill parents (COPMI) are at a high risk of developing severe mental illness (SMI) themselves and are likely to be the next generation of mentally ill patients (1). There are studies pointing to the fact, that the parental mental disorder launches a wave of risk factors that in turn predict the emergence of psychological problems in the offspring (2). Numerous studies have shown that a parental mental illness is a powerful risk factor for the development of a SMI in children (OR in the BELLA study of 2.4) (1, 3–8). Long-term studies were also able to show that COPMI have a higher life-time risk of developing SMI themselves (ranging from 41 to 77%) with subclinical symptoms emerging earlier and more often (5, 6). The treatment of the parental disorder has been associated with improved outcomes in COPMI (7, 9–13), although there are few studies on such effects (7, 14), and they typically target the same symptoms in the child as the parent's, while such specific transmission of disorders is not typical for COPMI (1). A recent meta-analysis (k = 9) by Cuijpers et al. (14) on the effects of psychological treatment of maternal depression on children's psychopathology resulted in an overall effect-size of g = 0.40. However, the studies included were very heterogeneous (k = 5 targeting women with post-partum depression; k = 4 targeting pregnant women or mothers of young children or mothers of children with psychological/psychiatric problems/disorders), and only 2 studies explicitly applied Cognitive Behavioral Therapy (CBT) as an intervention that resulted in an overall effect of g = 0.31 (14). An earlier meta-analysis by Siegenthaler et al. (15) on preventive interventions for children of mentally ill parents demonstrated a significant relative risk reduction of 40 % for the same disorder as the parents', and overall small effects for children's internalizing (g = −0.22) and externalizing (g = −0.16) symptoms. This analysis included interventions targeting children though and not specifically those that assessed parental psychotherapy effects on their children (15). Our own meta-analysis on preventive interventions for COPMI (16) resulted in effect sizes similar to those of Cuijpers et al. (14) for young (up to 5 years of age) children (g = 0.31), and overall smaller effects for older children (g = 0.14) that equal those of Siegenthaler et al. (15). Different longitudinal studies on parental anxiety and depressive disorders present heterogeneous effects of parental treatment on children. A 6 year prospective longitudinal study on the effects of parental panic treatment demonstrated that parental treatment is a significant predictor of children's anxiety symptoms (d = 0.49–1.09 for different parental psychopathology predictors) (7). The Sequenced Treatment Alternatives to Relieve Depression (STAR^*^D) Child study was designed to examine the relation between maternal remission from depression and children's functioning and psychopathology. The study demonstrated differential effects on child psychopathology in early, late, and non-remitting mothers, with early remission being associated with reduced child externalizing problems (~5% of Child Behavior Checklist (CBCL) externalizing symptoms explained) (13); similar results have been obtained in another large longitudinal study (9).

Studies focusing on the parent-child interaction tended to demonstrate poorer parenting skills in parents with mental disorders (17–20). The enhancement of such skills has been identified as a significant mediator in improving child outcomes (21). The Positive Parenting Program (Triple P) is a well-established program to enhance parenting skills in parents of children aged 0–16 years (Triple P Kids and Triple P Teens). Universal prevention effects have been established for the Triple P Kids program (22), as well as specific effects for child psychopathology (d = 0.473) (23, 24) that differ for mothers (d = 0.61) and fathers (d = 0.42). The effectiveness of the Triple P Teen program was also shown (25). Studies explicitly testing the parenting assumption in conjunction with the launch and grow assumption are lacking so far.

Regarding psychotherapy research, there have been suggestions that moving from an approach comparing an active treatment with a control group (“does it work?”) to one that examines putatively active treatments resulting in relative questions (“which works best?” or “how do the treatments differ?”) is advisable. If demand artifacts and differentiating non-specific from specific treatment factors are included, we arrive at this formula: Treatment A = E + D_T_ + T_NS_ + T_S(A)_ and Treatment B = E + D_T_ + T_NS_ + T_S(B)_ with E = all extraneous factors; D_T_ = demand characteristics treatment; T_NS_ = non-specific treatment factors; T_S_ = characteristic-specific treatment factors. Thus, the relative difference between the two active agents in the proposed study T_S(A)_ = CBT and T_S(B)_ = CBT+PPP estimates how the two treatments differ and enables us to determine relative effects (26). Such head-to-head studies on preventive interventions for COPMI assessing differential effects have been extremely rare; the classic format are “does it work?” studies (15).

Thus, we aim to implement a preventive intervention for COPMI with the aim of interrupting the transmission of mental disorders in children of a parent with a mental disorder. The preventive intervention is planned as a two-arm RCT to establish whether strengthening parenting skills results in incremental COPMI effects above and beyond state-of-the-art CBT for parents. Including an economic evaluation alongside the clinical trial, the results of this study will have an effect on the decision making process on resource allocation for this highly vulnerable group of children of mentally ill parents.

The RCT will thus target the following hypothesis: (1) the treatment of the parental disorder will result in improved child outcome (1st arm: CBT) (2, 7, 12), and (2) the parenting skills of parents with mental illness are impaired and enhancing such skills leads to better child outcomes, thus incremental effects will become apparent in the 2nd arm: CBT+Triple P (17, 18, 20, 27, 28).

Further research questions are: Is the clinical outcome associated with reduced direct medical, direct, and indirect non-medical costs? Are CBT and CBT+PPP associated with improved quality of life for parents and children? Are CBT and CBT+PPP associated with increased psychopathology knowledge of parents and children? Is CBT+PPP associated with higher parenting skills than CBT alone? Are the effects independent of type of diagnosis, comorbid disorders, and psychopharmacology?

## Methods

### Design

The planned study is a prospective, multicenter, confirmatory, randomized controlled phase III-trial with two parallel arms comparing the effects of state of the art CBT (control intervention), and CBT + Triple P (experimental intervention) for parents with a mental illness on their children. The study is coordinated by the Department of Psychology, Clinical Child-, and Adolescent Psychology at the Philipps University Marburg (UMR).

**Figure 1:**
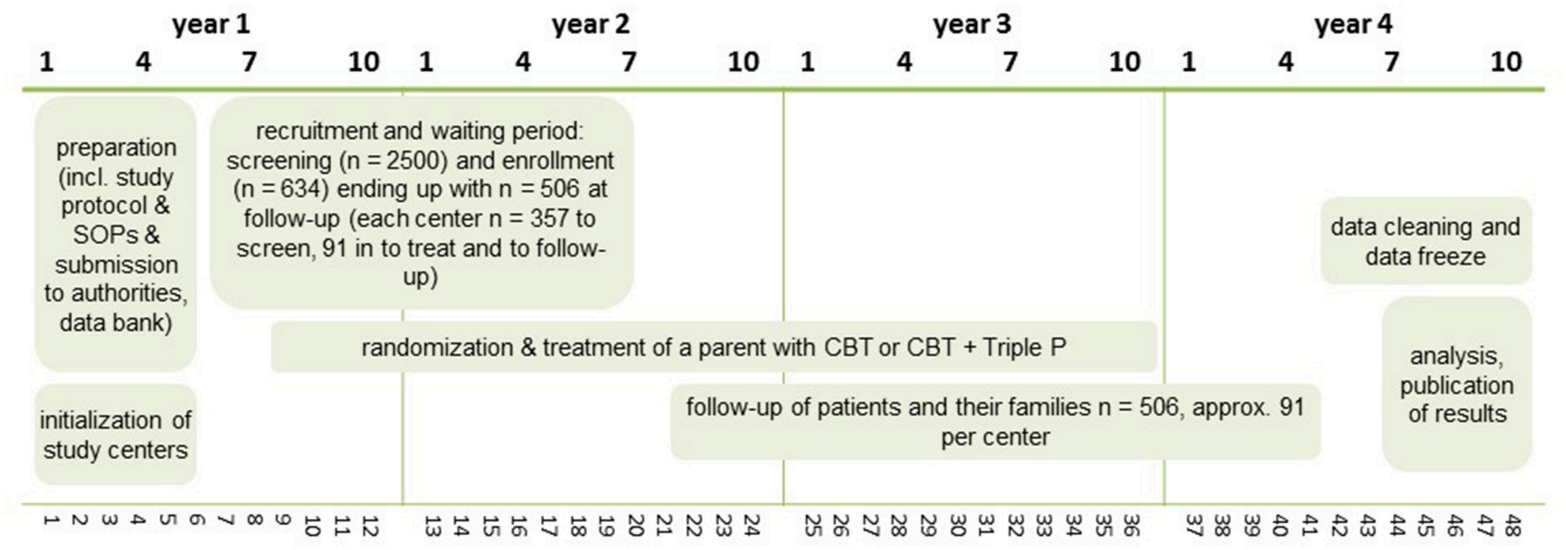
Study design.

After 3 months of study preparation (October–December 2017), a 15 months recruiting period has started in January 2018. The assessment period (first patient in until last patient out) will last for a total of 39 months. Six months are scheduled for data freeze, data cleaning and analysis. Thus, the duration of the whole trial is 48 months (see Figure 1).

**Figure 2:**
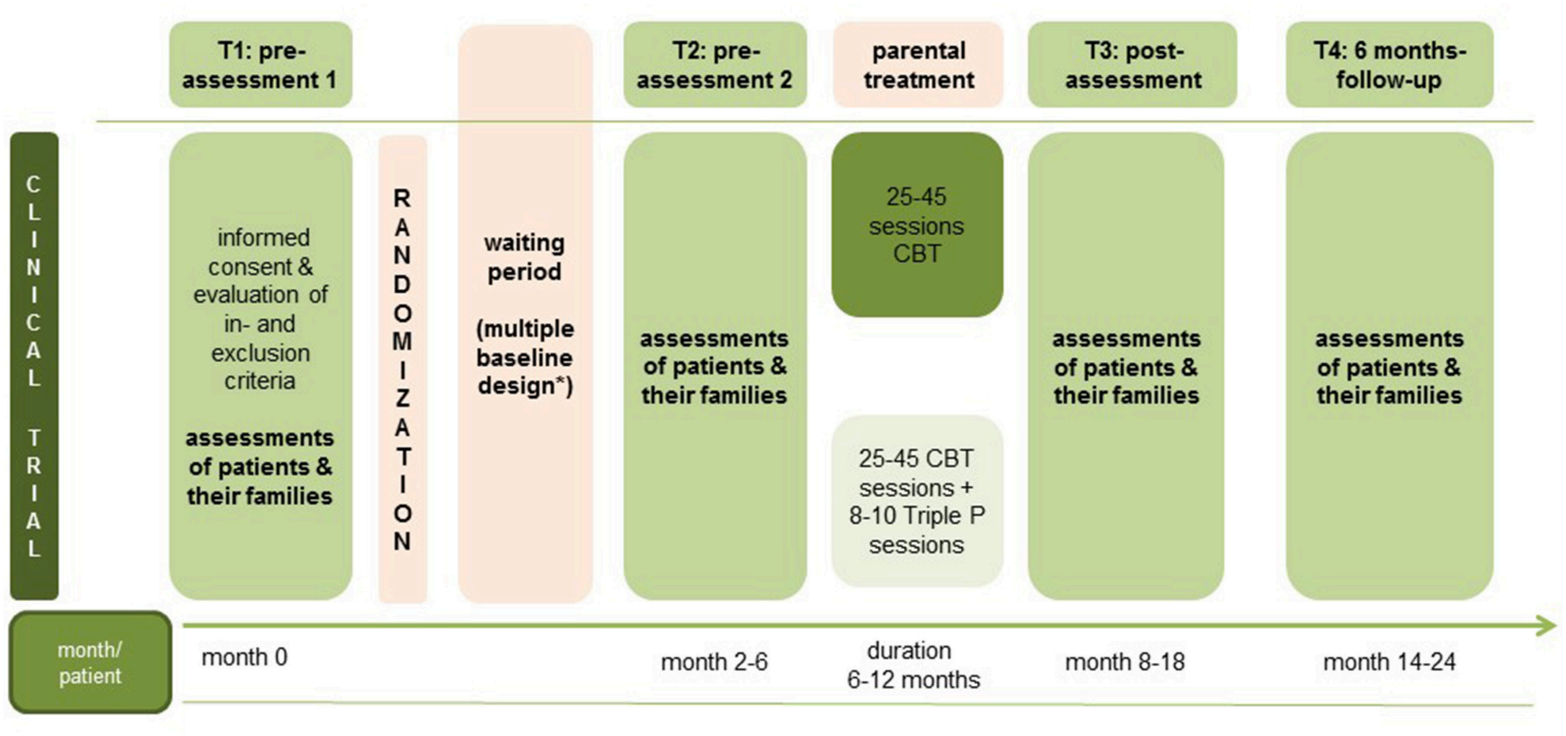
Study assessment points. *Waiting period can vary according to the regular waiting period in the study centers.

Using the same instruments (self- and other-report questionnaires as well as (clinical) interviews) each time, patients, and their families will be assessed at four main measurement points (see Figure 2): pre-assessment 1 (T1, beginning of waiting period for psychotherapy treatment (duration of waiting period depends on usual waiting period in the study center: multiple baselines), pre-assessment 2, (T2, beginning of parental psychotherapy), post-assessment (T3, after parental psychotherapy), follow-up-assessment (T4, 6 months after parental psychotherapy). Altogether, assessment time comprises between 4 and 5 h for each main assessment (T1, T2, T3, T4) for each family. Patients and therapists are also assessed after each treatment session for treatment fidelity and satisfaction. Every fifth session, the remission status of the patient is also assessed.

### Participants

A total of 634 parents with a mental illness (patients) shall be included in the study. The following criteria must be met for patients and their families to be included in the study: (1) patient seeks outpatient psychotherapeutic care, (2) patient currently meets diagnostic criteria for DSM-5 disorder (29), and (3) patient is caring for at least one child between the ages of 1.5–16 years. Patients and their families will not be included in the study if (1) patient is already in psychotherapeutic treatment, (2) patient needs acute inpatient treatment (e.g., acute risk of committing suicide or acute psychosis), (3) all children fulfill criteria for a severe mental illness and are in need of prompt treatment, (4) patient uses benzodiazepines continuously (intermittent drug use less than once every 2 weeks is allowed), or (5) family has insufficient German language skills. No further exclusion criteria will be applied. Hence, we seek to establish effects of parental psychotherapy on children in a naturalistic setting and want to specify the effects of the transgenerational transmission to test the launch and growth assumption. Patients in need of acute inpatient treatment are referred to cooperating hospitals. For children in need of prompt care, treatments are initiated at the study sites or the families are referred to other local specialists/psychiatric hospitals.

The study will be conducted at eight university outpatient clinics throughout Germany: Bielefeld, Bochum, Gießen, Landau, Leipzig, Mainz, Marburg, and Munich. All cooperating sites have established outpatient clinics for research and teaching as well as psychotherapy training institutes and are well-known specialized treatment centers for psychotherapy. The patients are primarily recruited from the university outpatient clinics at each study site. The study centers have continuous experience in conducting psychotherapy studies with recruitment of large patients numbers (30, 31). The patients to be recruited per center and fulfilling all inclusion criteria will be attainable over a 15-month acquisition period at all study sites without the need to change the existing infrastructure. If possible, all children between 1.5 and 16 years of age (at T1) and a partner living together with the family shall be included in the study. A legal guardian not living together with a child can upon request be offered to participate in the study and to complete questionnaires about the respective child. Inclusion only takes place if the family participates voluntarily in the study. There is no compensation for study participation.

### Interventions

All patients will receive between 25 (short-term) and 45 (long-term) weekly or bi-weekly sessions of individual state of the art Cognitive Behavioral Therapy (CBT). A high frequency therapy with more than one session per week is also allowed. Depending on the amount and the frequency of therapy sessions, the treatment will usually last between 6 and 12 months. The number of sessions and therapy duration will be documented, thus, allowing us to include therapy duration and number of sessions as a moderating factor in the analyses.

CBT can be considered the current “treatment of choice” for mental disorders in general (32). Central elements are psychoeducation that provides a framework for psychotherapy, cognitive components (i.e., debate of dysfunctional cognitions alias Beck), as well as behavioral interventions (i.e., exposure). As all study sites are outpatient clinics for research and teaching located at university psychology departments, they commit to the highest possible state of the art standard for CBT as outlined in “proceedings in psychotherapy/Fortschritte der Psychotherapie” of which Prof. Rief (Co-PI of COMPARE-family) is an editor (33). As the study shall be conducted as naturalistic as possible and a broad range of disorders shall be included, no specific study manual has been created, rather, the respective therapy manuals of the proceedings in psychotherapy will be used in this study.

Patients randomized to the experimental group will receive 8–10 additional group or individual sessions of the Positive Parenting Program (Triple P) parallel to the last third of the psychotherapy sessions to test whether the enhancement of parenting skills will result in improved child outcome, as suggested in different studies (17, 18, 20, 27, 28), above and beyond parental CBT. Triple P is a well-established, widely used and evidence-based program to enhance parenting skills. The Triple P Kids program can be used in groups or individual sessions with parents of children aged 0–12 years (34, 35) and the Triple P Teens program in groups with parents of children aged 12–16 years (36). The Triple P elements are: (1) promotion of a positive parenting style, (2) promotion of child development, (3) dealing with problem behavior, (4) behavior training sessions (34–36).

### Outcomes

#### Primary Outcome Measure

The primary outcome is the children's pathology according to the [(Caregiver-) Teacher Report Form (C-)TRF, caregiver/teacher version of the Child Behavior Checklist] score between baseline 2 (T2) and post treatment (T3) as well as between T2 and the 6-month follow-up (T4) after end of treatment (37, 38) as the literature shows that mentally ill parents can be biased in rating their children's symptoms (39). Further, teacher ratings have been shown to demonstrate greater predictive validity in the long term ratings (40). In comparison to shorter questionnaires [e.g., Strengths and Difficulties Questionnaire (SDQ) (41)], the CBCL also produces more variance. As the CBCL is used in various studies as a primary outcome measure (15), we will be able to compare our data to a broad range of other results. Further outcome measures are mental disorders according to the Diagnostic Interview of Mental Disorders for parents and children (DIPS and Kinder-DIPS) (42–44) respectively for children under the age of 6 years the Structured Interview for Preschool Ages (SIVA) (45) and the parent rating of the children's pathology according to the CBCL (for detailed descriptions see below). Thus, we will be able to validate the teacher ratings with the Kinder-DIPS diagnoses and can compare the concordance of the teacher and parent ratings.

#### Secondary Outcome Measures

Children's and parents dimensional severity index of a broad range of mental disorders according to the DSM-5 based clinical interview (Kinder-)DIPS (42–44) respectively the SIVA (45), parent's psychopathology according to the Brief Symptom Inventory (BSI) (46), personality traits (PID-5-BF) (47), children's psychopathology according to the CBCL-parentversion (37, 38), parenting skills (EFB) (48), parental stress (ESF) (49), children's and parents knowledge about mental disorders [semistructured interview adapted from Beardslee and Röhrle, (50)], direct medical costs and direct and indirect non-medical costs (TiC-P) (51), health-related quality of life in parents [EuroQoL/EQ-5D-5L, AQoL-8D (52, 53)], health-related quality of life in children (KIDSCREEN-10) (54, 55) (for details see Table 1).

**Table 1:** List of diagnostic domains and measurements.

	**Who?**	**Where?**	**T1**	**T2**	**Intermediate**	**T3**	**T4**
Eligibility screening	Family	Local	x				
Socio-demographics	Parents	Local	x				
Structured clinical interview with the patient (DIPS)	Patient	Local	x	x		x	x
Structured clinical interview for children (Kinder-DIPS/SIVA)	Parent	Local	x	x		x	x
Brief Symptom Inventory (BSI)	Parents	Online	x	x	x (patient)	x	x
Personality traits (PID-5-BF)	Parents	Online	x	x		x	x
Child Behavior Checklist (CBCL)	Parents	Online	x	x		x	x
(Caregiver-) Teacher Report Form [(C-)TRF, caregiver/teacher version of the CBCL]	Teacher	Online	x	x		x	x
Parenting Skills (EFB)	Parents	Online	x	x		x	x
Parental Stress (ESF)	Parents	Online	x	x		x	x
Knowledge about mental disorders	Parents, children	Local	x	x		x	x
Direct and indirect costs in children and parents (adapted TiC-P)	Parents	Online	x	x		x	x
Health-related quality of life in parents (EuroQoL/EQ-5D-5L, AQoL-8D)	Parents	Online	x	x		x	x
Health-related quality of life in children (KIDSCREEN-10)	Parents, children (>8)	Online	x	x		x	x
(Serious) adverse events	Parents, children	Local		x	x (patient)	x	x
Psychopharmaka and other drugs	Parents, children	Local	x	x	x	x	x
Concomitant interventions	Parents, children	Local	x	x	x	x	x

### Instruments

The (Kinder-) DIPS is a diagnostic interview for mental disorders and is available for the diagnoses of mental disorders from age 6 to adulthood according to ICD-10 and DSM-5. The SIVA is a diagnostic interview for mental disorders for preschool ages according to ICD-10 and DC: 0–5. A translation table to DSM-5 diagnoses has been created for this study. For parents we use the DIPS; for their children the Kinder-DIPS or SIVA. Conducting the interviews with the patient takes around 60–90 min as well as the child assessment with the Kinder-DIPS or SIVA for each child. Different studies have shown good quality criteria for these interviews (43, 45).

The Achenbach System of Empirically Based Assessment (ASEBA) (56) consists amongst others of questionnaires for preschool (C-TRF and CBCL 1,5-5, consisting each of 100 problem items) and school-ages (TRF and CBCL, consisting each of 113 problem items). Answers are rated on a three point Likert-scale (0 = Not true to 2 = Very True or Often True) and can be scored on different subscales, the second order scales internalizing problems and externalizing problems as well as on a total problem scale. Studies have reported good to very good internal consistency with *r* > 0.86 for the second order scales and the total problem scale of the preschool age-versions (37) and Cronbach's alpha >0.80 for the second order scales and at least.93 for the total problem scale in the school age-versions (38). In the current study we use the parent (CBCL) and teacher (TRF) ratings of the ASEBA.

The Brief Symptom Inventory (BSI) is a self-report questionnaire consisting of 53 items that are rated on a five point Likert-scale (0 = not at all to 4 = very much). Answers are scored on nine Primary Symptom Dimensions and three Global Indices. Internal consistency with Cronbach's alpha > 0.70 for the subscales and > 0.90 for the Global Indicee GSI is good to very good (46).

The Personality Inventory for DSM-5-Brief Form (PID-5-BF) is a self-report questionnaire assessing 5 personality traits (negative affect, detachment, antagonism, disinhibition, and psychoticism). The 25 items are rated on a 4 point Likert-scale (0 = very false or often false to 3 = very true or often true). An average score for each domain and an overall score can be calculated with higher scores indicating greater dysfunction. The average domain and overall personality dysfunction scores were found to be reliable in the DSM-5 Field Trials (47).

The Elternstressfragebogen (ESF) is a German self-report questionnaire with 38 items assessing parental stress. The answers are rated on the four scales Parental Stress, Role Restriction, Social Support, and Partnership. The internal consistency is good with Cronbach's alpha > 0.76 (49).

The Erziehungsfragebogen (EFB) is the German adaptation of the English Parenting Scale (57). The self-report questionnaire consists of 35 items and can be rated on the scales Overreactivion, Laxness, and Verbosity as well as on a total score. The internal consistencies of the scales Overreaction, Laxness, and the total score is acceptable to good with Cronbach's alpha > 0.74. For Verbosity the internal consistency is lower with Cronbach's alpha > 0.59 (48).

To assess knowledge about mental disorders, a semi structured interview was adapted from Beardslee and Röhrle (50). Domains of the interview are knowledge about mental disorders in general, knowledge about the primary diagnosis of the mentally ill parent, causes of the mental illness, coping with the mental illness and communication. Conducting the interviews separately with all family members over the age of 6 years takes 15 min each.

The questionnaire on healthcare consumption and productivity losses for patients with a Psychiatric disorder (TiC-P) is a comprehensive and widely used self-report questionnaire focusing on establishing direct medical costs and indirect costs (e.g., productivity losses due to absenteeism and presenteeism) (51). The TiC-P is a feasible and reliable instrument for collecting data on medical consumption and productivity losses in patients with common mental health conditions (58). The TiC-P has been previously adapted for use in Germany (59). For this study, we adapted the TiC-P for use in children.

The EQ-5D-5L is a widely used instrument to calculate quality-adjusted life years (QALYs) (52). The EQ-5D-5L comprises 5 items covering 5 domains (mobility, self-care, usual activities, pain/discomfort, and anxiety/depression), each of which is rated as causing “no problems,” “slight problems,” “moderate problems,” “severe problems,” and “extreme problems.” Theoretically, the EQ-5D-5L generates 3,125 different health states. Preference-based utilities for each of these health states are available for Germany with “full health” and “death” being anchored at 1 and 0, respectively (60).

The Assessment of Quality of Life (AQoL) instruments are health-related multi-attribute utility quality of life instruments. The AQoL is a reliable and valid instrument (53). The AQoL-8D consists of five psycho-social and three physical dimensions. With one exception (dimension “senses”), each of these represents a psychometrically valid sub-scale [e.g., tests indicate they measure a common construct (61)]. Utility scores were obtained by a four-stage methodology (62).

The KIDSCREEN-10 index consists of 10 items each answered on a 5-point Likert scale. The index provides a good discriminatory power along the health-related quality of life trait-continuum. The KIDSCREEN-10 Index shows good psychometric properties (63). Utility scores will be derived by an algorithm for mapping the KIDSCREEN-10 index onto the CHU9D utility scores, a preference-based instrument developed specifically for application in cost-utility analyses (54).

### Sample Size

The sample size calculation is based on the primary outcome measure (change in TRF score between T2 and T3) which is hierarchically ranked on top of the multiple testing procedure. Based on the results described above (14, 15, 64), it is assumed that the standardized treatment effect for this outcome expressed by Cohen's d amounts to d = 0.25. With a two-sided significance level of α = 0.05 and a power of 1–β = 0.8 using a two-sample *t*-test, 253 patients per group (*n* = 506 in total) are required. Since the primary outcome is measured on the child level and it is possible to enroll multiple children per patient, a hierarchical multi-level model with patients at level 1 and children at level 2 will be fitted. Taking a drop-out rate of 20 % into account, *n* = 634 patients need to be enrolled into the trial in the analysis. This attrition rate is conservative and based on results from the longitudinal STAR^*^D study that also carried out long-term follow-up assessments (13), as well as on other studies in the field that report even lower attrition rates (65, 66). The problem of attrition and missing values will also be addressed in the analysis by applying the intention-to-treat principle. For missing values, imputation techniques will be applied, thus partly resolving this problem. Since it is assumed that parts of the outcome variance can be explained by the inclusion of covariates, the actual power of the analysis by a linear multi-level model is expected to be higher than 1–β = 0.8. Assuming that the number of children enrolled per patient amounts to 1.5, we expect that *n* = 950 children will participate in the trial. The enrollment of more than one child per patient is expected to yield an additionally increased power. Sample size calculation was performed using SAS v9.4 (SAS Institute, Cary, NC).

### Randomization

Patients will be assigned in a 1:1 ratio to either the control intervention (CBT) or the experimental intervention (CBT+Triple P) through a centralized web-based tool (www.randomizer.at). Randomization will be performed stratified by center, comorbidity (yes/no), and total number of children (1/more than 1). To achieve equal group sizes per stratum block randomization will be performed. The block length will be defined by the study biometrician and treated confidentially to prevent selection bias.

### Blinding

Assessment interviews will be conducted and analyzed by clinician-raters blinded to the treatment condition. Raters at post and follow-up assessments must not be the therapist of the particular patient being assessed and analyzed.

### Statistical Methods

Our primary efficacy analysis will be based on the full analysis set (FAS) according to the intention-to-treat (ITT) principle, reflecting the recommendations given in relevant guidelines (67). The FAS is defined to include all patients enrolled who are assigned to the treatment group they were originally randomized to, regardless of whether they actually underwent the assigned treatment or not. This will be the primary population for evaluating all efficacy endpoints and subject characteristics. Additionally, the per-protocol (PP) population, including all FAS patients with no major protocol deviations, will serve as a secondary analysis population and be used for sensitivity analyses. Before lock of the database, each patient's allocation to the FAS or PP population will be defined in the statistical analysis plan.

The two hypotheses to be assessed in the primary efficacy analysis are ordered hierarchically: In the first step, the null hypothesis ${\text{H}}_{0}^{\text{I}}$: $\mu_{\text{CBT}+\text{PPP}}^{\text{T}3-\text{T}2}$ = $\mu_{\text{CBT}}^{\text{T}3-\text{T}2}$ for the primary outcome “change in teacher CBCL (Teacher Report Form/TRF) score between T2 and T3” is tested at the two-sided significance level of 5% against the alternative ${\text{H}}_{1}^{\text{I}}$: $\mu_{\text{CBT}+\text{PPP}}^{\text{T}3-\text{T}2}$ ≠ $\mu_{\text{CBT}}^{\text{T}3-\text{T}2}$. If ${\text{H}}_{0}^{\text{I}}$ can be rejected, the null hypothesis ${\text{H}}_{0}^{\text{II}}$: $\mu_{\text{CBT}+\text{PPP}}^{\text{T}4-\text{T}2}$ = $\mu_{\text{CBT}}^{\text{T}4-\text{T}2}$ for the second primary endpoint “change in TRF score between T2 and T4” is tested at the two-sided level of 5% against its alternative ${\text{H}}_{1}^{\text{II}}$: $\mu_{\text{CBT}+\text{PPP}}^{\text{T}4-\text{T}2}$ ≠ $\mu_{\text{CBT}}^{\text{T}4-\text{T}2}$. Application of this multiple test procedure for a priori ordered hypotheses ensures control of the family-wise type I error rate at a level of 5%. The null hypotheses will be assessed using a linear mixed multi-level model with patients at level 1 and children at level 2, adjusting for center, number of comorbidities, number of children, baseline TRF score at T2, and length of waiting period between T1 and T2 (in weeks).

Data missing for the primary outcome variable will be replaced by using multiple imputation (68) which takes the covariates treatment group, center, number of comorbidities, number of children, baseline TRF score at T2, and length of waiting period between T1 and T2 (in weeks) into account by applying the fully conditional specification method (69). This will be realized using the option “FCS” of the SAS “MI” procedure implemented in SAS 9.4. Sensitivity analyses will be performed by applying alternative methods dealing with missing data such as complete case analysis. All secondary outcomes will be evaluated descriptively, and descriptive *p*-values are reported together with 95% confidence intervals for the corresponding effects. Further exploratory analyses will be performed to identify potential prognostic factors (e.g., parental disorder, child psychopathology, socio-economic status) and mediators (e.g., Brief Symptom Inventory/BSI, Parenting Questionnaire/EFB, Parental Stress Inventory/ESF) for an intervention effect. The safety analysis includes calculation of frequencies and rates of adverse and serious adverse events together with 95% confidence intervals. All analyses will be performed with SAS version 9.4 or higher.

The health-economic evaluation will involve a combination of a cost-effectiveness analysis (CEA) and a cost-utility analysis (CUA) (70, 71). The economic evaluation will be done from a societal perspective (all relevant costs) and a public health care perspective (only direct medical costs) within a 6 month time frame. In the CEA, the incremental cost-effectiveness ratio (ICER) will be expressed as the incremental costs per point improvement on the primary clinical outcome (CBCL scores in children; parents' BSI scores). In the CUA, the ICER will be expressed as incremental costs per quality-adjusted life year (QALY) gained as based on the EQ-5D-5L (52, 72) (parents) and KIDSCREEN-10 (63, 73) (children). Sampling uncertainty in the ICER will be handled using non-parametric bootstrapping by resampling patient-level data to generate 2,500 simulations of the ICER. We will bootstrap the SURE model (seemingly unrelated regression equations; sureg command in Stata) to allow for correlated residuals of the cost and effect equations (74). Ninety five percent confidence intervals (CI) will be obtained by the bootstrap acceptability method, since parametric techniques are inappropriate for use on skewed variables and ratios (75).The bootstrapped ICERs will be plotted in a cost-effectiveness plan where the horizontal axis reflects differences in effects and the vertical axis differences in costs. The bootstrapped ICERs will also be shown in a cost-effective acceptability curve disclosing the probability that the intervention is cost-effective for a range of willingness-to-pay ceilings (76). To test the robustness of the base-case findings, a probalistic sensitivity analysis will be done. Several assumptions made in the base-case scenario will be changed to assess their impact on the ICER (e.g., QALY calculation based on AQoL-8D).

### Data management, monitoring, and quality assurance

#### Training of Study Personal and Treatment Fidelity

All study personal (assessors, therapists, and supervisors) will be trained before taking part in the study. As all sites are part of associated university training institutes that conform to the highest psychotherapeutic standards. All therapists will receive intense training in CBT for the different disorders. The Triple P institute in Münster, which is the official and certified Triple P institution in Germany, will carry out Triple P training. Trained and certified supervisors will supervise every fourth treatment session at the study sites. At each participating center, a clinical project manager is responsible that the study is conducted in accordance to the procedures outlined in the study protocol. Concerning all information and data collected during the study, all study personal maintain professional secrecy and confidentiality.

After each treatment session, the therapists indicate CBT adherence by rating a checklist extracted from the disorder specific therapy manuals as outlined in the proceedings in psychotherapy (33). Upon termination of a study therapy, a supervisor rates the overall adherence of the therapy based on the adherence checks completed after every session. The adherence rate is documented in the eCRF for further analyzes.

Treatment fidelity/integrity will be analyzed with rating schemes for 5% randomly selected videotaped treatment sessions by the study coordination at the UMR. Before the videotapes are rated; they will be checked for any cues/hints potentially indicating the treatment condition; those will be erased.

#### Data Collection and Retention

For each included family, a study file will be created at the study site in which all local study documents will be archived. An electronic case report form (eCRF) will be used for the data collection using the secure web based electronic data capture (EDC) tool REDCap (77). The participants complete most of the questionnaires directly in the eCRF. A study staff at each site creates log-in-codes that are handed out to the participants during on site sessions with which the participants (parents, children over the age of 8 years and caregivers/teachers) can log into REDCap to complete the questionnaires at home. The families are asked to fill out the forms independently. The eCRF is programmed to not allow skipping answers to prevent missing data. If questions occur, filling out the eCRF can be paused and continued later on with a trained assessor at a study site. A study staff will enter locally collected data into REDCap preferably on the day of data collection. Upon termination of the study, all local study documents at the study sites will be sent to the UMR where the documents will be digitalized. For supervision and to ensure treatment fidelity, all study sessions will be videotaped. The video data will be stored in encrypted form using VeraCrypt which is useable free of cost under the Apache License 2.0. All videotapes will be send to the study coordination at the UMR and stored there. Upon termination of the study, all video data will be destroyed.

All local study documents are part of the Investigator Site File (ISF) as outlined in section 8 of the ICH Consolidated Guideline which is stored and archived according to the legal retention period. Data quality assessment of the eCRF will be done continuously by the Institute of Medical Biometry and Informatics Heidelberg during the study. Any entry and correction in the EDC system will be documented automatically in an audit file. Completeness, validity, and plausibility of data will be checked in time of data entry (edit-checks) and using validating programs which will generate queries. All assessments and modifications are immediately accessible via web access. The clinical project manager at each study site is also responsible for online transmission of data and site-specific quality assurance. Upon completion of the study, all data will be exported into different data formats for further analyses. The primary data will be made accessible to the public for re- and meta-analyses through PsychData at the Leibniz Institute for Psychology Information (https://www.psychdata.de/). All data will be used in accordance to data protection regulations and the data safety guidelines of the German Psychological Society (DGPs).

#### Ethical and Legal Aspects

Working with a highly vulnerable group, the procedures set out in this study protocol are designed to ensure that the investigators abide by the principles of the Good Clinical Practice (GCP) guidelines of the International Conference on Harmonization (ICH) and the Declaration of Helsinki and its later supplements. The study will be carried out adhering to local legal requirements. The lead ethics committee at the department of psychology at the UMR has approved the study procedure, study information, and informed consent forms in December 2017 and its subsequent amendments in May and July 2018. A positive ethics committee vote is required at a study site (ethics committees at the departments of psychology at Bielefeld University, Ruhr-University Bochum, Justus Liebig University Giessen, University of Koblenz-Landau, University of Leipzig, Johannes Gutenberg University Mainz, and Ludwig-Maximilians-University Munich), before the inclusion of a first patient at the respective site. At the beginning of the study, patients and their families are informed verbally and written about the aims of the study and the study procedures. All patients and their families (children over 6 years of age) need to provide written informed consent for study participation. For children to participate in the study, written informed consent by all legal guardians is required. Every participant can drop out of the study at any time. All dropouts will be documented in the eCRF.

#### Pseudonymization

After signing the informed consent, each study participant is assigned a pseudonymized screening-ID. The screening-ID consists of three parts: specific number for the study site, consecutive screening number and individual coding number (1 = patient, 2 = partner, 3, 4, 5 = included children in order of increasing age, X = ex-partner). Members of a family can be recognized by the first two parts of the screening-ID (specific study site number and consecutive screening number).

There is a coding list on paper at each study site on which names and screening-IDs are documented. The coding lists, which are kept locked away, are accessible only to study personal. The lists will be destroyed upon termination of the study but no later than 2021/12/31. After that, all data will be fully anonymized. As long as the coding lists exist, participants can request the deletion, respectively the destruction of all their collected data.

#### Clinical Monitor

A clinical monitor is responsible for overviewing the implementation of the study in accordance to the ICH-GSP guidelines at the study sites. The clinical monitoring includes pre-study, initiation, intermediate and close-out visits to the study sites. During the site visits, the clinical monitor examines the protocol adherent study implementation, the safety of the patients and the data consistency (e.g., comparison of local study documents and eCRF records).

#### Data Safety Monitoring Board

An independent Data Safety Monitoring Board (DSMB), consisting of a university professor, which is an expert in the field of the study, and a biometrician, is responsible for monitoring the study as well as assessing the study protocol adherence and the study progress (especially the recruiting plan). The DSMB will be informed about all safety aspects of the study (especially serious adverse events, SAE) and will review them regularly. If necessary, the DSMB will recommend changes to the study protocol or the termination of the study.

### Dissemination

The results of this study will be made available to the larger scientific community via peer-reviewed publications in open access scientific journals. Politicians, public health services and stakeholders will be informed through the COMPARE website, conferences, teaching seminars, flyers, newsletters, and personal contacts throughout the study and beyond, thus improving public policy and health care decisions concerning preventive interventions and treatments for COPMI. As all study sites are university based outpatient clinics the implementation of results in ongoing and future practice is ensured.

Upon termination of the study, all primary data will be made available to the scientific community in a completely anonymized manner for re- and meta-analyses using PsychData, a data-sharing platform developed by the Leibniz Institute for Psychology Information (ZPID, https://www.psychdata.de).

## Detailed Study Procedure

**Figure 3:**
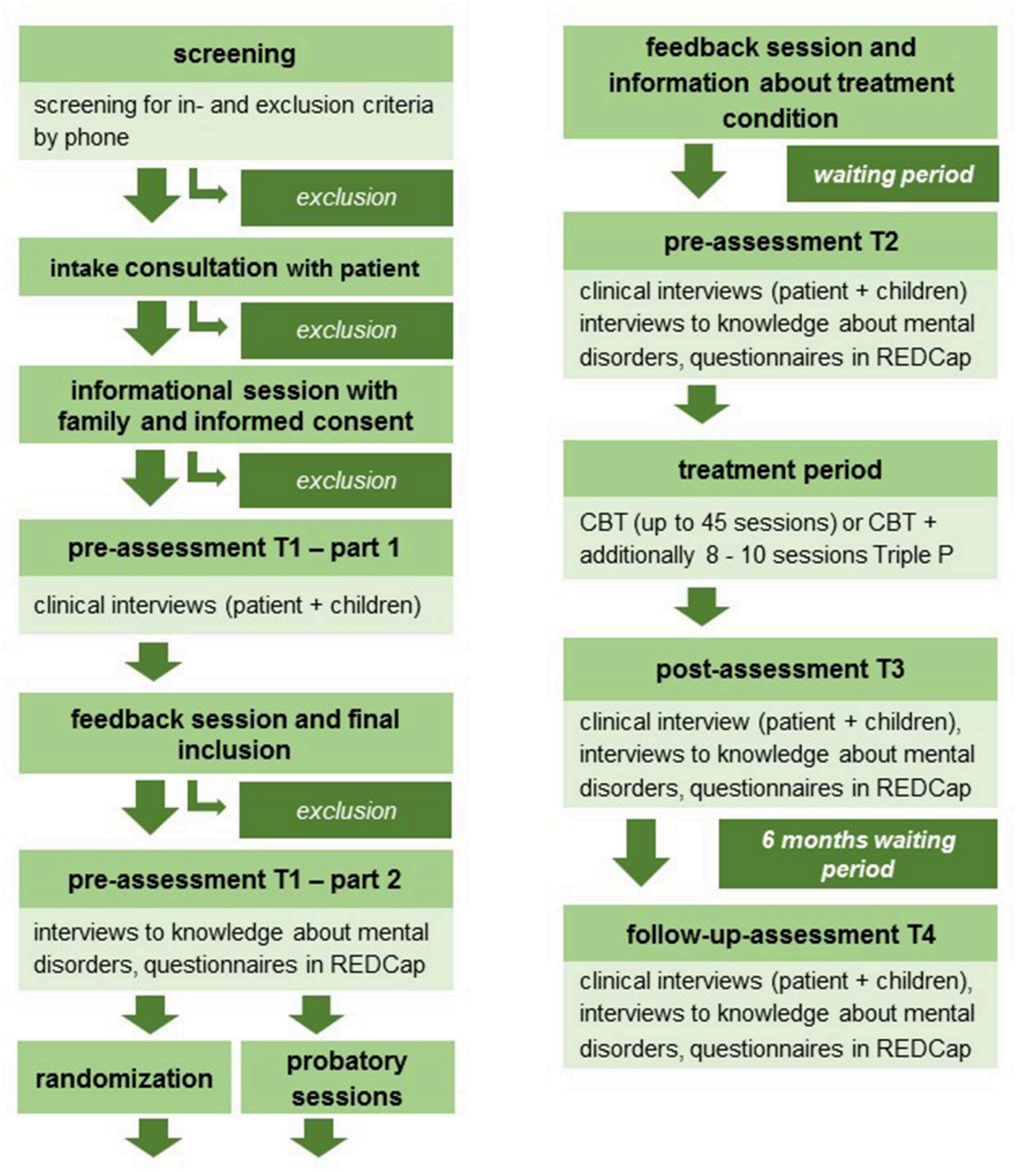
Study flowchart.

A detailed flowchart of the study procedure is presented in Figure 3.

### Pre-assessment T1 and Assessment of Eligibility

After an initial screening of eligibility for an as early and effective as possible selection of patients and their families and a regular intake consultation with the patient in the outpatient clinic, the patients and their families (children over the age of 6 years) are invited to an informational session at the study center. During this session, the families are informed about the study procedure before signing their consent to participate in the study. For pseudonymized data collection, each participant is assigned a Screening-ID. To assess a broad range of DSM-5 based psychological disorders and comorbid ones as well as severity ratings, structured clinical interviews for patients (DIPS), and children (Kinder-DIPS parent version or SIVA) are conducted with the patient respectively a parent (for each child separately). The parent, with whom the Kinder-DIPS/SIVA is conducted, has to be the same for all children of a family at all measurement points. The in- and exclusion criteria are checked on the basis of the information collected during the screening and the diagnoses based on the clinical interviews. Feedback to diagnoses is given in a separate session. If a patient and its family fulfill all the in- and none of the exclusion criteria, the family is included in the trial and records for the eCRF are created for all the included family members. The family is invited to another session in which interviews about knowledge about mental disorders are conducted with each included family member. After this session codes are handed out with which the family members can log into REDCap at home to complete the questionnaires. Forms with REDCap-codes for the children's caregivers or teachers (one caregiver/teacher for each included child) are also handed out to the family with the request to forward them to the respective caregiver/teachers. There is no reference to the clinical trial of the COMPARE-family project on the caregiver/teacher's form; rather, the questionnaire is generally presented as part of a study at the department of psychology the UMR. After the final inclusion in the study, the randomization to one of the two treatment arms (CBT or CBT+Triple P) takes place. If needed, further probatory sessions can take place at the study centers. In a separate feedback session, the patient is informed about the treatment condition and results of the diagnostic process.

### Pre-assessment T2

After the usual waiting period for a psychotherapy treatment in the study centers (waiting period can differ between the study centers depending on their usual waiting periods for a psychotherapy treatment), structured clinical interviews for patients and children are conducted again with the patient respectively the same parent as in T1 (for each child separately). The family is invited to another session in which interviews about knowledge of mental disorders are conducted with each included family member, and codes for logging into REDCap at home to complete the questionnaires are handed out. The forms with REDCap-codes for the children's caregivers/teachers are also handed out to the family with the request to forward them to the respective caregivers/teachers.

### Treatment Period

All patients receive between 25 to 45 sessions of individual CBT. Patients allocated to the experimental intervention (CBT+Triple P) receive in addition 8 to 10 group or individual Triple P sessions parallel to the last third of the CBT sessions. After every therapy session, patients, and therapists complete a session-feedback screener including aspects of therapeutic alliance and symptom intensity, as well as on fidelity. (Serious) adverse events [(S)AE] are also assessed after every therapy session with a checklist. If a (S)AE occurs, a report has to be filed and send to the study coordination at the UMR. Every fifth session, the patients are also handed out a code for logging into REDCap at home to complete the BSI to assess remission status (early, late, and non-remitters).

### Post-assessment T3

Using the same measurements as in the pre-assessments, the post-assessment is conducted by an assessor who is blind to the treatment condition. Structured clinical interviews for patients and children are conducted again with the patient, respectively the same parent as in T1 (for each child separately). During a separate session, interviews on knowledge about mental disorders are conducted with each included family member separately, (S)AE are assessed for every included family member, and codes for logging into REDCap at home to complete the questionnaires are handed out at the end. The forms with REDCap-codes for the children's caregivers/teachers are also handed out to the family with the request to forward them to the respective caregivers/teachers. If a (S)AE occurs, a report has to be filed and sent to the study coordination at the UMR.

### Follow-Up-Assessment T4

After a waiting period of 6 months, the follow-up-assessment is conducted by a blind assessor in the same manner as the post-assessment. The structured clinical interviews for patients and children are conducted again with the patient respectively the same parent as in T1 (for each child separately). In a separate session, knowledge about mental disorders and (S)AE are assessed for each included family member separately. At the end of this session, codes for logging into REDCap at home to complete the questionnaires are handed out. The forms with the code for the children's caregivers/teachers are also handed out to the family with the request to forward them to the respective caregivers/teachers. If a (S)AE occurs, a report has to be filed and sent to the study coordination at the UMR.

## Discussion

We expect that in arm I (CBT), the parents will improve through treatment as will their children (2, 7, 12). Since, parenting skills of parents with a mental illness are likely impaired and enhancing such skills leads to better child outcomes (17, 18, 20, 27, 28), we expect that additional incremental effects will become apparent in arm II (CBT+Triple P). We will use the Positive Parenting Program (Triple P) in addition to CBT, as Triple P is an evidence-based, widely used, and well-established program. The positive effects of Triple P have been demonstrated in parents and children (22, 23, 78), although incremental effects above and beyond parental CBT have not been researched in conjunction with COPMI so far.

The established magnitude of this specific incremental effect will result in precise recommendations for this high-risk group that will impact clinical practice (i.e., practice parameters, treatment guidelines). As outlined above, COPMI are most likely to constitute the next generation of patients with a mental illness, and we assume that this intervention will contribute to the prevention of SMI in this specific high-risk group.

As studies so far have focused primarily on specific disorders (e.g., anxiety, depression) and have excluded comorbidities (1, 3), though comorbidities actually occur with the most patients, the COMPARE study will fill this research gap by including a broad range of mental disorders and by not excluding comorbidities. A double baseline measurement with multiple baselines depending on the usual waiting periods in the study centers (T1: 1st assessment of parents and children via clinical interviews and questionnaires; T2: 2nd assessment with the same instruments) will enable us to assess parental disorder effects on the child that can then be related to parental treatment effects (T3: after completion of 25 short-term to 45 long-term sessions of state-of-the-art CBT or CBT+TripleP) for a 6-month follow-up (T4). As there are differential parental psychotherapy effects on children depending on the parental remission status (early, late, and non-remitters), we will also assess such effects (9, 13). By including different therapy durations (short- vs. long-term therapy) we will be able to shed light on treatment duration effects.

### Research and Clinical Implications

The RCT is part of the COMPARE consortium [see Christiansen et al. (79) in this research topic] with the subprojects COMPARE-emotion, COMPARE-interaction, COMPARE-work, and COMPARE-school. The findings of the Compare consortium will not only allow for the estimation of parental treatment and parenting skill effects on their children, but also for the novel approach of testing the core assumptions of the transgenerational transmission of mental disorders model (5) comprehensively. The cost-effectiveness and cost-utility analyses from a societal and a health care system perspective will be a basis for negotiations with health care providers, as COPMI at risk can currently not receive professional help, except if they have already developed disorders themselves. The evidence of this trial will hopefully contribute to an understanding of preventing the development of disorders in COMPI to reach this goal. The identification of specific risk profiles will contribute to such an improved understanding and will result in tailored interventions that might either be more preventive or interventive in character, depending on the individual risk.

### Limitations

COMPARE-family plans four extensive assessments for all family members as well as up to 55 therapy assessments. Even though the majority of patients has positive attitudes toward extensive diagnostic assessments (80), this might also be a burden on the family, especially as the add-on projects require additional assessments at the same assessment times.

The implementation of a self-wait control design in the RCT was originally planned with a waiting period of 6 months between the first assessment of the patients and their families and the beginning of the therapy. Due to the new psychotherapy guidelines in Germany, that became effective in April 2017, the waiting periods to receive psychotherapy treatment have decreased enormously at the study sites. Although the time span to receive a place in a treatment program can still be up to 9 months, many of the recruiting outpatient clinics have tried to cut those time spans down (e.g., 1–3 months waiting periods). With the original 6 months waiting period, patients wanting to participate in the COMPARE study would consequently have had to wait longer for their psychotherapy treatment than necessary. For the study centers this not only created ethical but also practical issues with interested patients preferring treatments with shorter waiting periods to the participation in COMPARE. With the now implemented multiple baseline design, the waiting period can vary between the study centers depending on the usual waiting periods on-site, therefore not disadvantaging study participants.

The observation period of only 39 months in total is rather short for capturing effects on the prevention of SMI, but due to funding, a longer observation period could not be implemented. For assessing long-term effects, a follow-up study is already planned and all participants are asked permission to be contacted again later on.

The majority of outpatient clinics will most likely treat patients with depressive and affective disorders, a fact possibly hampering the aim of the COMPARE study to cover a broad range of disorders. However, as all outpatient clinics are university-based and all recruiting centers have different research foci, chances are good that the range of disorders will be represented. This will also raise the generalizability of findings.

## Author Contributions

HC, MS, and KG contributed to conception and design of the study. CB drafted the health economic evaluation study. MK, CK, and JK organized the database and performed the statistical analyses. MS wrote the first draft of the manuscript. All authors contributed to sections of the manuscript, manuscript revision, read, and approved the submitted version.

### Conflict of Interest Statement

The authors declare that the research was conducted in the absence of any commercial or financial relationships that could be construed as a potential conflict of interest.
